# Supplementary material for: A national study of burnout and spiritual health in UK general practitioners during the COVID-19 pandemic
Source: PLoS One. 2022 Nov 2;17(11):e0276739. doi: 10.1371/journal.pone.0276739 (PMC9629610; doi:10.1371/journal.pone.0276739)
Supplement: S1 Table — (DOCX) [file pone.0276739.s001.docx]

Table S1: Comparison of burnout classification and spiritual score: adjusted multinomial regression

| **Burnout Classification** | Unadjusted Relative risk ratio  [95% confidence  interval] |
| --- | --- |
|  |  |
| **Lowest Risk of Burnout** |  |
| **Spiritual Health** |  |
| Low spiritual health score | 0.25 [0.11-0.57] |
| Moderate spiritual health score | 1 |
| High spiritual health score | 3.89 [2.63-5.75] |
| **Gender** |  |
| Woman | 1 |
| Man | 1.61 [1.15-2.25] |
| **Ethnic Group** |  |
| White |  |
| Asian or Asian British | 1.08 [0.66-1.76] |
| Black, Mixed or other group | 1.25 [0.64-2.47] |
| **Religion** |  |
| No religion | 1 |
| Humanist | 2.30 [0.72-7.31] |
| Religion | 1.44 [1.01-2.04] |
| **Years working as a GP** |  |
| 0-5 | 1 |
| 6-10 | 0.80 [0.44-1.47] |
| 11-20 | 1.09 [0.64-1.84] |
| 20-30 | 1.22 [0.72-2.06] |
| 30+ | 1.89 [1.00-3.56] |
| **Number of sessions worked as a GP per week** |  |
| 1-4 | 1.10 [0.72-1.67] |
| 5-7 | 1 |
| 8-9 | 1.57 [0.79-3.13] |
| 10+ | 1.07 [0.36-3.14] |
| **Area of Primary Medical Qualification** |  |
| UK | 1 |
| European Economic Area graduate | 2.29 [1.15-4.56] |
| International Medical Graduate (IMG) | 0.98 [0.50-1.89] |
| **Intermediate risk of burnout** | Comparator |
| **Highest risk of burnout** |  |
| **Spiritual Health** |  |
| Low spiritual health score | 5.09 [3.33-7.78] |
| Moderate spiritual health score | 1 |
| High spiritual health score | 0.31[0.14-0.68] |
| **Gender** |  |
| Man | 1.21 [0.85-1.72] |
| Woman | 1 |
| **Ethnic Group** |  |
| White | 1 |
| Asian or Asian British | 1.56 [0.99-2.46] |
| Black, Mixed or other group | 0.91 [0.40-2.04] |
| **Religion** |  |
| No religion | 1 |
| Humanist | 0.93 [0.20-4.20] |
| Religion | 0.88 [0.62-1.24] |
| **Years working as a GP** |  |
| 0-5 | 1 |
| 6-10 | 1.55 [0.89-2.73] |
| 11-20 | 1.18 [0.69-2.03] |
| 20-30 | 0.96 [0.54-1.69] |
| 30+ | 0.63 [0.26-1.53] |
| **Number of sessions worked as a GP per week** |  |
| 1-4 | 0.89 [0.56-1.40] |
| 5-7 | 1 |
| 8-9 | 1.18 [0.54-2.57] |
| 10+ | 1.10 [0.37-3.24] |
| **Area of Primary Medical Qualification** |  |
| UK | 1 |
| European Economic Area graduate | 0.77 [0.27-2.21] |
| International Medical Graduate (IMG) | 0.54 [0.23-1.26] |
