# Supplementary material for: A national study of burnout and spiritual health in UK general practitioners during the COVID-19 pandemic
Source: PLoS One. 2022 Nov 2;17(11):e0276739. doi: 10.1371/journal.pone.0276739 (PMC9629610; doi:10.1371/journal.pone.0276739)
Supplement: S2 Table — (DOCX) [file pone.0276739.s002.docx]

#### Table S2- classifications of burnout risk for analysis used for this study

| Classification of overall burnout score | Depersonalisation | Emotional Exhaustion | Personal Accomplishment |
| --- | --- | --- | --- |
| Highest | High | High | Low |
| Intermediate | High  High  High  Moderate  Moderate  Moderate  Low  Low  Low | High  Moderate  Low  Moderate  High  Moderate  High  Moderate  Low | High or moderate  High or moderate or low  High or moderate or low  High or moderate or low  High or moderate or low  High or moderate or low  High or moderate or low  High or moderate or low  Moderate or low |
| Lowest | Low | Low | High |
