# Supplementary material for: A national study of burnout and spiritual health in UK general practitioners during the COVID-19 pandemic
Source: PLoS One. 2022 Nov 2;17(11):e0276739. doi: 10.1371/journal.pone.0276739 (PMC9629610; doi:10.1371/journal.pone.0276739)
Supplement: S3 Table — (DOCX) [file pone.0276739.s003.docx]

#### Table S3: Burnout Tertile ‘cut offs’ used in analysis of this data, with cut offs used in literature for comparison. ^1^

| Tertile cut off | Depersonalisation  (“high”commonly >10) | Emotional Exhaustion  (“high”commonly >27) | Personal Accomplishment  (“low” commonly <33) |
| --- | --- | --- | --- |
| High | 19.5 | 45.8 | 42.4 |
| Moderate | 9.8 | 34 | 35.7 |
| Low | 3.2 | 19 | 27.2 |

1. Doulougeri K, Georganta K and Montgomery A. “Diagnosing” burnout among healthcare professionals: Can we find consensus? *Cogent Medicine* 2016; 3: 1. DOI: 10.1080/2331205X.2016.1237605.
