# Supplementary material for: A national study of burnout and spiritual health in UK general practitioners during the COVID-19 pandemic
Source: PLoS One. 2022 Nov 2;17(11):e0276739. doi: 10.1371/journal.pone.0276739 (PMC9629610; doi:10.1371/journal.pone.0276739)
Supplement: Attachment — Submitted filename: plosone response to reviewers 23rd July.docx [file pone.0276739.s004.docx]

**Response to Reviewers**

We would like to thank the two reviewers who made thoughtful and useful suggestions for our paper titled “A National Study of Burnout and Spiritual Health in UK General Practitioners During the COVID-19 Pandemic”.

Please find our responses to each of the requested changes below.

*1. Please ensure that your manuscript meets PLOS ONE's style requirements, including those for file naming. The PLOS ONE style templates can be found at* 
[*https://journals.plos.org/plosone/s/file?id=wjVg/PLOSOne_formatting_sample_main_body.pdf*](https://journals.plos.org/plosone/s/file?id=wjVg/PLOSOne_formatting_sample_main_body.pdf) *and* [*https://journals.plos.org/plosone/s/file?id=ba62/PLOSOne_formatting_sample_title_authors_affiliations.pdf*](https://journals.plos.org/plosone/s/file?id=ba62/PLOSOne_formatting_sample_title_authors_affiliations.pdf)

Thank you for these clear instructions, the manuscript has been re-formatted.

*2. Please provide additional details regarding participant consent. In the ethics statement in the Methods and online submission information, please ensure that you have specified what type you obtained (for instance, written or verbal, and if verbal, how it was documented and witnessed). If your study included minors, state whether you obtained consent from parents or guardians. If the need for consent was waived by the ethics committee, please include this information.*

*Once you have amended this/these statement(s) in the Methods section of the manuscript, please add the same text to the “Ethics Statement” field of the submission form (via “Edit Submission”).*

*For additional information about PLOS ONE ethical requirements for human subjects research, please refer to* [*http://journals.plos.org/plosone/s/submission-guidelines#loc-human-subjects-research*](http://journals.plos.org/plosone/s/submission-guidelines#loc-human-subjects-research)*.*

Apologies for this omission-“Written consent was sought online as a condition for proceeding with the survey” has been added to the methods and the ethics statement has been revised.

*3. In your Data Availability statement, you have not specified where the minimal data set underlying the results described in your manuscript can be found. PLOS defines a study's minimal data set as the underlying data used to reach the conclusions drawn in the manuscript and any additional data required to replicate the reported study findings in their entirety. All PLOS journals require that the minimal data set be made fully available. For more information about our data policy, please see* [*http://journals.plos.org/plosone/s/data-availability*](http://journals.plos.org/plosone/s/data-availability)*.*

*Upon re-submitting your revised manuscript, please upload your study’s minimal underlying data set as either Supporting Information files or to a stable, public repository and include the relevant URLs, DOIs, or accession numbers within your revised cover letter. For a list of acceptable repositories, please see* [*http://journals.plos.org/plosone/s/data-availability#loc-recommended-repositories*](http://journals.plos.org/plosone/s/data-availability#loc-recommended-repositories)*. Any potentially identifying patient information must be fully anonymized.*

*Important: If there are ethical or legal restrictions to sharing your data publicly, please explain these restrictions in detail. Please see our guidelines for more information on what we consider unacceptable restrictions to publicly sharing data:* [*http://journals.plos.org/plosone/s/data-availability#loc-unacceptable-data-access-restrictions*](http://journals.plos.org/plosone/s/data-availability#loc-unacceptable-data-access-restrictions)*. Note that it is not acceptable for the authors to be the sole named individuals responsible for ensuring data access.*

*We will update your Data Availability statement to reflect the information you provide in your cover letter.*

This is human research participant data. Participants were asked to consent to anonymised data “becoming part of a data set which can be accessed by other users running other research studies at Newcastle University and in other organisations.  These organisations may be universities, or NHS organisations. [This] information will only be used by organisations and researchers to conduct research.” The authors are concerned that this doesn’t include consent for public data sharing, only for further research in universities or NHS organisations. Data will be shared upon reasonable request to the authors. The sentence “Data Access: While participants were not consented to allow public sharing of this data, data is available upon reasonable request to the authors.“ has been added. The authors have noted the difficulties here, and will amend the consent process for the future, to allow data sharing more easily.

*4. Your ethics statement should only appear in the Methods section of your manuscript. If your ethics statement is written in any section besides the Methods, please move it to the Methods section and delete it from any other section. Please ensure that your ethics statement is included in your manuscript, as the ethics statement entered into the online submission form will not be published alongside your manuscript.*

The ethics statement has been moved to the Methods section, and the previous ethics statement has been deleted.

*5. Please include captions for your Supporting Information files at the end of your manuscript, and update any in-text citations to match accordingly. Please see our Supporting Information guidelines for more information:* [*http://journals.plos.org/plosone/s/supporting-information*](http://journals.plos.org/plosone/s/supporting-information)*.*

This has been amended


Reviewers' comments:

Reviewer's Responses to Questions

**Comments to the Author**

1. Is the manuscript technically sound, and do the data support the conclusions?

The manuscript must describe a technically sound piece of scientific research with data that supports the conclusions. Experiments must have been conducted rigorously, with appropriate controls, replication, and sample sizes. The conclusions must be drawn appropriately based on the data presented.

Reviewer #1: Partly

Reviewer #2: No

2. Has the statistical analysis been performed appropriately and rigorously?

Reviewer #1: Yes

Reviewer #2: No

3. Have the authors made all data underlying the findings in their manuscript fully available?

The [PLOS Data policy](https://eur03.safelinks.protection.outlook.com/?url=http%3A%2F%2Fwww.plosone.org%2Fstatic%2Fpolicies.action%23sharing&data=05%7C01%7Corla.whitehead%40newcastle.ac.uk%7C0464a65dd70d43f16f6a08da495984c5%7C9c5012c9b61644c2a91766814fbe3e87%7C1%7C0%7C637902946340781336%7CUnknown%7CTWFpbGZsb3d8eyJWIjoiMC4wLjAwMDAiLCJQIjoiV2luMzIiLCJBTiI6Ik1haWwiLCJXVCI6Mn0%3D%7C3000%7C%7C%7C&sdata=Ih1C0LhvWtvsGN%2BfvPaO%2FQVVPtSVfx812H%2BSfnyde04%3D&reserved=0) requires authors to make all data underlying the findings described in their manuscript fully available without restriction, with rare exception (please refer to the Data Availability Statement in the manuscript PDF file). The data should be provided as part of the manuscript or its supporting information, or deposited to a public repository. For example, in addition to summary statistics, the data points behind means, medians and variance measures should be available. If there are restrictions on publicly sharing data—e.g. participant privacy or use of data from a third party—those must be specified.

Reviewer #1: No

Reviewer #2: No

4. Is the manuscript presented in an intelligible fashion and written in standard English?

PLOS ONE does not copyedit accepted manuscripts, so the language in submitted articles must be clear, correct, and unambiguous. Any typographical or grammatical errors should be corrected at revision, so please note any specific errors here.

Reviewer #1: Yes

Reviewer #2: Yes

*5. Review Comments to the Author

Please use the space provided to explain your answers to the questions above. You may also include additional comments for the author, including concerns about dual publication, research ethics, or publication ethics. (Please upload your review as an attachment if it exceeds 20,000 characters)*

*Reviewer #1: This paper addresses an important issue and has relevant findings. I present my concern for its methodology and presentation here:

Table 1: what is the difference between “no religion” and “atheist” here? Where will the agnostics (sometimes estimated to be 15% of the UK population) go? Given the very important role of this variable in this entire study, a better explanation of these categories is needed and more discussion is needed if some of frequencies deviate too much from previously estimated frequencies in comparable populations.*

Participants were asked “what is your religion?” and to choose from “No religion, Christian, Muslim, Jewish, Sikh, Buddhist, Humanist, Hindu, or Other (please write).” This was left open to the participant to interpret, as to whether this is a cultural, ethnic, philosophical, or religiosity label. This study looks at spiritual health (which often involves religious aspects) as defined by the population being studied, and while religious identification was asked as a potential confounding variable, it was not the variable under investigation.

Those who wrote in the “other” box were either left as “other” where they described being of mixed religious background, or from a smaller religious group. Those who named a subgroup of the main religions were analysed as part of that main religion. The authors debated how to analyse Humanists, as in a previous survey by the authors participants regarded Humanism as ‘a religion’, yet others stated Humanism is a lack of religion, hence the analysis of the religion variable being conducted with the small number of Humanists excluded. The nature of quantitative work in an area that is so dependent on personal philosophy is challenging, as in many ways we are looking to measure the immeasurable.

*Line 197: so “atheists” are included as one of the religions???*

The nature of atheism is also debatable- in our study, atheists were included in ‘no religion’, as were agnostics unless they named a religion they were agnostic about. There is an argument, put by a participant, that atheism is “stronger” than “no religion”, and is a belief system in itself. “Atheists were included in ‘no religion.’” has been added to the text to clarify where atheists were placed. “or atheist” has been added at line 207.

*Table format is fairly unconventional in this paper and Table 4 starts to get confusing. I suggest that the authors take a look at the published tables in PLOS series journals to better follow the expected style of presenting a multinomial logistic regression. Moreover, readers will expect to see what are the covariates used in the multinomial logistic regression, at least in the footnotes to Table 4 . Tables need to be self-explanatory, without having the readers go back to the text to check what are the covariates used.*

Thank you for your feedback on table 4. Lines 232-233 are the footnote which gives the covariates used. We believe the table is self-explanatory. The supplementary table (table S1) gives all unadjusted relative risks. As the main research question for this paper is regarding any association between the spiritual health score, and the burnout score, the regression analysis presented aims to answer that question.

*As spirituality could mean very different things for religious people and non-religious people, I suggest that the multinomial model be run among the religious and then among the non-religious, separately. In other words, using religion as an effect modifier rather than a confounder will make more sense here. Alternatively, the authors could create an interaction term that multiplies the spirituality variable with the binary religion variable.*

We agree, spirituality means different things for many groups of people. The authors spent time considering the spiritual health scores and believe that the FACIT-Sp-NI is equally applicable to those of any degree of religiosity. This study doesn’t measure religiosity, and the religion question was left open for interpretation for the participant, and therefore could well be part of ethnic or cultural identification rather than active religious participation. While a longer survey could have ‘drilled down’ into religiosity, religious practice, cultural membership of a faith, etc, the survey would likely not have the response rate it did if it had. The outcome of interest was the spiritual health score, the FACIT-Sp-NI, which does include a ‘faith’ subdomain in the score. The questions in the faith subdomain refers to ‘faith or spiritual beliefs’, rather than religiosity, and was included in the total FACIT-Sp-NI score analysed. Data were gathered around religious identification to identify any sampling bias towards only those with a religious identification for example. Authors previously have argued that spiritual health and religiosity overlap so much as to be the same (eg. Harold Koenig), however the definition we use, given by the population under study (UK GPs) in a previous survey, describes religious and spiritual behaviour as one aspect of overall spiritual health, similarly to Puchalski et al’s consensus definition. The data in table 3 does show that those who named a religion had significantly higher spiritual health scores than those who didn’t, which would be expected given the faith subdomain. Burnout scores were not statistically significantly affected by identification with a religion. (Table 3) As the FACIT-Sp-NI does have a faith subdomain, this could be analysed as a measure of religiosity, however the research question is regarding general spiritual health, and we have deliberately drawn no conclusions about religiosity as an effect modifier- this study is asking about the wider concept of spiritual health. Previous studies have addressed religiosity and burnout, finding mixed results.

The religion variable measures the respondent’s identification with a specific faith group, which is helpful. Yet, this measurement does not get to the level of religiosity, i.e., how important is this faith to the individual, frequency of religious practice/service, etc. This aspect of religiosity could influence mental health outcomes more directly rather than the categories of faith groups or the binary classification of religion vs. no-religion, and thus should be discussed as a limit in Discussion under the context of previous findings.

Thank you, “While the spiritual score included a faith domain, religiosity was not assessed.” has been added in the strengths and limitations section for clarity. In our study, we did not measure religiosity.

Reviewer #2: Thank you very much for the opportunity to review the manuscript entitled “A National Study of Burnout and Spiritual Health in UK General Practitioners During the COVID-19 Pandemic”. GP burnout during COVID-19 is a profound challenge that needs to be addressed. However, I am afraid that I have the following concerns about the novelty of the study topic, soundness of research design, and depth of discussion:

1. Introduction: the authors stated that “Previous studies on the relationship between spiritual health and burnout have been vulnerable to response or sampling bias, used unvalidated instruments, analysed single domains of burnout or spiritual health in isolation, and conflated religion, and wider spiritual health” (p4 lines 88-91). As far as I understand, this statement is closely linked to the novelty of this study. However, I am concerned about two issues. First, the statement was made without any reference. Second, this study also suffered from some limitations such as response or sampling bias, analyzed spiritual health in isolation.

Thank you for highlighting these, references for the statement have been added. The sentence has been changed to “Previous studies on the relationship between spiritual health and burnout have been vulnerable to response or sampling bias, used unvalidated instruments, analysed single domains of burnout or single domains of spiritual health in isolation.” to clarify that other studies have looked at single domains (eg. Spiritual activities, use of spiritual coping, etc) rather than the use of a validated broad spiritual measure like the FACIT-Sp-NI.

*2. Another issue that is closely related to the novelty of this study is the theoretical/practical importance of understanding the relationship between spiritual wellness and burnout. However, I am afraid that I did not find strong justification in the introduction section.*

Thank you for highlighting this. “Identifying whether spiritual health and burnout are related in UK GPs will potentially allow a novel view of research into organisational and individual interventions to improve GPs spiritual health, possibly mitigating the current workforce crisis.” This sentence has been added to the introduction.

*3. The authors stated that “While burnout in GPs has been quantified using the MBI-HSS previously, this study adds an up to date quantification of burnout levels during the heart of the pandemic, using robust scores.” (p5 lines 93-95). I agree that quantification of burnout levels amongst GPs during pandemic is critical to help understand the psychological consequence of COVID-19. However, the prevalence/degree of burnout was neither part of the research objective “aiming to generate robust data to better understand relationships between practitioner health, wellbeing and burnout” (p5 lines 99-100), nor discussed in depth with prior studies that investigated burnout during COVID-19.*

As far as the authors are aware, there have been no other national GP surveys in the UK using the ‘gold standard’ measure of burnout in populations, the Maslach Burnout Inventory – Human Services Survey. Jefferson et al^1^’s systematic review found one survey using the Perceived Stress Scale (not a burnout measure), and was limited to Leicester, rather than a national survey.^2^ Reference to Jefferson et al’s systematic review has been added to the comparison with other literature: “ A systematic review of GP burnout during the pandemic found two European studies that found 24.5% to 46.1% GPs had emotional exhaustion.”

*4. Participant recruitment: it seems that the authors adopted convenience sampling technique to include GPs who had worked between March 2020 and May 2021. What were the start and end dates of data collection? Besides the sample representativeness issues, a year seems to be very long for a cross-sectional study, considering that there were several waves of COVID during 2020 – 2021.*

The survey was conducted during April and May 2021. (line 48)

*5. Statistical analysis: the responses of burnout do not seem to be in normal distribution (Figure 1). In this case, I do not suggest reporting mean values or conducting t test/ANOVA. I did not quite understand why not to control for areas of current work in regression analysis.*

As the sample was large, it was felt that the type 1 error using t test and ANOVA would be insignificant. To remedy any concerns, the table has been re-written to contain median values, IQR and used the Mann-Whitney and Kruskal-Wallis tests.

There was no reason to hypothesise that burnout scores during this period would vary by broad locality currently worked in. There was no statistically significant difference in burnout scores by locality currently worked in. This data was gathered to ensure an adequate spread of recruitment, from all four nations within the UK. There were more participants from England, being the largest of the nations, and therefore comparing the smaller nations to England, could have led to misleading/erroneous conclusions by the reader about which area has GPs at lower/higher risk of burnout.

*6. Discussion: I wish I could get more insights from the findings by comparing them with prior studies that investigated burnout during COVID-19.*

This would be ideal, however the novelty of our study is that there aren’t similar published studies investigating burnout using validated scores in this population during COVID-19. We have added a sentence comparing our findings with those in Jefferson et al’s systematic review: “This study does not show that gender affects risk of being at high risk of burnout, in contrast with other studies identified in a recent systematic review, which found female doctors more at risk of stress.”

*[NOTE: If reviewer comments were submitted as an attachment file, they will be attached to this email and accessible via the submission site. Please log into your account, locate the manuscript record, and check for the action link "View Attachments". If this link does not appear, there are no attachment files.]

While revising your submission, please upload your figure files to the Preflight Analysis and Conversion Engine (PACE) digital diagnostic tool,* [*https://pacev2.apexcovantage.com/*](https://eur03.safelinks.protection.outlook.com/?url=https%3A%2F%2Fpacev2.apexcovantage.com%2F&data=05%7C01%7Corla.whitehead%40newcastle.ac.uk%7C0464a65dd70d43f16f6a08da495984c5%7C9c5012c9b61644c2a91766814fbe3e87%7C1%7C0%7C637902946340781336%7CUnknown%7CTWFpbGZsb3d8eyJWIjoiMC4wLjAwMDAiLCJQIjoiV2luMzIiLCJBTiI6Ik1haWwiLCJXVCI6Mn0%3D%7C3000%7C%7C%7C&sdata=UwCUdSk%2FU8344nfxqYD2LowxKOzogXWfMnbLyq9QhG8%3D&reserved=0)*. PACE helps ensure that figures meet PLOS requirements. To use PACE, you must first register as a user. Registration is free. Then, login and navigate to the UPLOAD tab, where you will find detailed instructions on how to use the tool. If you encounter any issues or have any questions when using PACE, please email PLOS at* [*figures@plos.org*](mailto:figures@plos.org)*. Please note that Supporting Information files do not need this step.*

1.	Jefferson L, Golder S, Heathcote C, et al. GP wellbeing during the COVID-19 pandemic: a systematic review. *British Journal of General Practice* 2022; 72: e325. DOI: 10.3399/BJGP.2021.0680.

2.	Trivedi N, Trivedi V, Moorthy A, et al. Recovery, restoration, and risk: a cross-sectional survey of the impact of COVID-19 on GPs in the first UK city to lock down. *BJGP Open* 2021; 5: BJGPO.2020.0151. DOI: 10.3399/BJGPO.2020.0151.
